# Use of an automated blood culture system (BD BACTEC™) for diagnosis of prosthetic joint infections: easy and fast

**DOI:** 10.1186/1471-2334-14-233

**Published:** 2014-05-04

**Authors:** Angela M Minassian, Robert Newnham, Elizabeth Kalimeris, Philip Bejon, Bridget L Atkins, Ian CJW Bowler

**Affiliations:** 1 Department of Microbiology, Oxford University Hospitals NHS Trust, John Radcliffe Hospital, Headley Way, Headington, Oxford OX3 9DU, UK; 2 Bone Infection Unit, Nuffield Orthopaedic Centre, Windmill Road, Oxford OX3 7LD, UK

**Keywords:** BACTEC™, Prosthetic joint infection, Culture, *Propionibacteria*

## Abstract

**Background:**

For the diagnosis of prosthetic joint infection (PJI) automated BACTEC™ blood culture bottle methods have comparable sensitivity, specificity and a shorter time to positivity than traditional cooked meat enrichment broth methods. We evaluate the culture incubation period required to maximise sensitivity and specificity of microbiological diagnosis, and the ability of BACTEC™ to detect slow growing *Propionibacteria spp*.

**Methods:**

Multiple periprosthetic tissue samples taken by a standardised method from 332 patients undergoing prosthetic joint revision arthroplasty were cultured for 14 days, using a BD BACTEC™ instrumented blood culture system, in a prospective study from 1st January to 31st August 2012. The *“gold standard”* definition for PJI was the presence of at least one histological criterion, the presence of a sinus tract or purulence around the device. Cases where > =2 samples yielded indistinguishable isolates were considered culture-positive. 1000 BACTEC™ bottle cultures which were negative after 14 days incubation were sub-cultured for *Propionibacteria spp.*

**Results:**

79 patients fulfilled the definition for PJI, and 66 of these were culture-positive. All but 1 of these 66 culture-positive cases of PJI were detected within 3 days of incubation. Only one additional (clinically-insignificant) *Propionibacterium spp.* was identified on terminal subculture of 1000 bottles.

**Conclusions:**

Prolonged microbiological culture for 2 weeks is unnecessary when using BACTEC™ culture methods. The majority of clinically significant organisms grow within 3 days, and *Propionibacteria spp.* are identified without the need for terminal subculture. These findings should facilitate earlier decisions on final antimicrobial prescribing.

## Background

The diagnosis of prosthetic joint infection (PJI) in the routine microbiology laboratory has historically been labour-intensive, requiring daily inspection of enrichment broths such as Robertson’s cooked meat. Semi-automated methods may provide easier and more rapid alternatives. Some routine laboratories are now using instrumented blood culture systems such as BD BACTEC™ for the incubation of various sterile site specimens. This continuous detection system eliminates the need for daily inspection and the requirement for terminal subculture. We previously demonstrated that this method was as sensitive and as specific as using Robertson’s cooked meat broth (sensitivity 87%, 95% confidence interval (CI) 72–100, vs. 83%, CI 66–99; specificity 98%, CI 96–100, vs. 97%, CI 95–100) and more sensitive than using Fastidious Anaerobic broth (sensitivity 57%, CI 35–78), compared to a gold standard of histology for diagnosis of PJI [1].

Previous work on samples from 284 patients undergoing hip and knee revisions suggests that prolonging culture beyond 7 days is required [2]. 26% of organisms were isolated only after 7 days of culture, especially *Propionibacteria spp*. Two more recent studies have similarly suggested prolonged incubation for optimal recovery of *Propionibacterium acnes* from periprosthetic samples [3,4]. However, these studies were performed using traditional manual enrichment broth/solid agar culture methods, only sub-culturing when broths were cloudy or after a fixed time period. We evaluate the duration of incubation required for the diagnosis of PJI using the BD BACTEC™ instrumented blood culture system.

## Methods

This was a prospective laboratory study over a 7-month period (January to August 2012).

Tissue samples originating from patients with suspected PJI were identified on arrival in the laboratory. These samples had been taken using a standardised method [5]. Information was obtained on the site of sampling, clinical features, antibiotic therapy at the time of sampling, number of samples sent per patient and histological analysis. Time to culture-positivity for both aerobic and anaerobic BACTEC™ cultures of samples was also recorded.

### Definition of infection and culture positivity

All patients over the study period who underwent a revision arthroplasty for suspected infection met the definition for “suspected PJI”. For evaluation of Sensitivity (Sn) and Specificity (Sp) of BACTEC™, the definition of *“gold standard infection”* included positive histological analysis for infection AND/OR clinical criteria: the presence of a sinus tract from skin to the prosthesis OR frank pus identified adjacent to the prosthesis at operation. These are in line with definitions of PJI in recent guidelines from the Infectious Diseases Society of America [6]. Cases where 2 or more samples yielded indistinguishable isolates were considered “culture-positive”. Further analyses were carried out using a less stringent criterion for culture positivity, where cases with a single sample positive for a virulent organism were also considered “culture-positive”. All other cases were considered “culture-negative”.

Histological significance was determined by the degree of infiltration by neutrophil polymorphonuclear leukocytes as outlined in previous studies [7-9], which have shown that the presence of at least five neutrophils per high-power field is strongly correlated with PJI and significant bacteriological growth. Quantitative cell counts of synovial aspirates were not routinely done so do not form part of our definition of PJI.

Organisms of the same species were deemed indistinguishable if they had the same colonial morphology, the same biochemical features as determined by the biochemical profiles generated from appropriate API kits (bioMerieux Vitek Inc., Hazelwood, Mo.), and an identical extended antibiotic susceptibility pattern.

### BACTEC™ method

Samples were sent at room temperature in sterile universal containers from the operating theatre directly to the laboratory. Transport times were monitored for a subset of 250 of the 713 samples. Of these 75% were processed with 6 hours of sampling, and 90% within 20 hours. Samples awaiting processing were refrigerated at 4°C. On arrival samples were processed as previously described [1]. Briefly, samples were disrupted to release bacteria by adding 3 ml of sterile saline and sterile glass beads (Equine and Ovine laboratories) with vigorous shaking (vortexing) for 15 seconds at 40 Hertz. 0.5 mL of sample was inoculated (via a safety device Vacutainer) into a BD BACTEC™ Lytic/10 Anaerobic/ F bottle, and 0.5 mL inoculated into a BACTEC™ Plus Aerobic/F bottle. These were incubated for 14 days. For those bottles which flagged positive, a Gram stain was performed and an inoculum sub-cultured. If no organisms were seen on Gram stain, after subculture the BACTEC™ bottle was returned to the BACTEC™ 960 machine for further monitoring. If there was no growth on subculture, this was recorded. BACTEC™ bottles were monitored until the end of the 14 day incubation. All isolates from positive bottles were identified on the basis of growth characteristics, antibiotic sensitivity and biochemical profiles (API, bioMerieux Vitek Inc., Hazelwood, Mo.).

### Sub-culturing of negative samples

We selected 1000 consecutive BACTEC™ bottle cultures which had not “flagged” positive at the end of their 14-day incubation period. These bottles were terminally sub-cultured onto fastidious anaerobic agar (FAA) and lysed blood “chocolate” agar. The FAA culture plates were incubated in an anaerobic jar and the chocolate plates incubated in a CO_2_ incubator at 37°C. Both plates were read after 5 days incubation and all bacteria isolated were identified on the basis of growth characteristics, antibiotic sensitivity and biochemical profiles (API, bioMerieux Vitek Inc., Hazelwood, Mo.).

### Statistical analysis

Data were analysed using STATA version 10 (Stata Corp., College Station, TX, USA). Our main analyses were based on patients (cases), incorporating data on all culture-positive cases combined with all culture-negative cases over the same time-frame. We calculated sensitivity (Sn), specificity (Sp), positive and negative predictive values (with Binomial exact 95% confidence intervals) for each of increasing culture incubation periods, starting from 1 day. Receiver Operator Characteristics (ROC) analysis was used to identify the time-point giving the best combination of sensitivity and specificity for BACTEC™.

This report is an analysis of data from routine laboratory processing of samples taken for routine healthcare. No research related contact with human subjects occurred. Our institution, the Oxford University Hospitals National Health Service Trust, classifies our work as a service evaluation and therefore did not require a submission for ethical review.

## Results

### Epidemiology of cases with “gold standard infection”

A total of 455 bacterial isolates were grown from 1328 samples taken from 332 patients with suspected PJI. 79 patients met the definition of *“gold standard infection”*. For these patients a mean number of 4 samples per patient were received. 47 were male, 32 female and the mean age was 69 years. 50 patients had infection relating to hip joint prostheses, 21 knee, 5 ankle and 3 shoulder joint prostheses.

53 of the 79 patients (67.1%) met the histological criteria for infection, 1 was equivocal, 6 were negative and 19 had no histological analysis performed. 62 (78.5%) in total met one or more clinical criteria; of these, 31 had frank pus adjacent to the prosthesis at the time of operation, 18 had a sinus communicating with the prosthesis and 4 had both pus and a sinus. The remaining 9 patients were included as infected on clinical grounds due to the presence of a deep collection communicating with the joint at surgery (albeit not frank pus). 22 patients (27.8%) met clinical criteria alone, although for 19 of these there was no histological analysis of samples performed. 17 (21.5%) patients met the histological criterion for infection alone and 40 (50.6%) patients met *both* clinical and histological criteria for infection.

**Figure 1:**
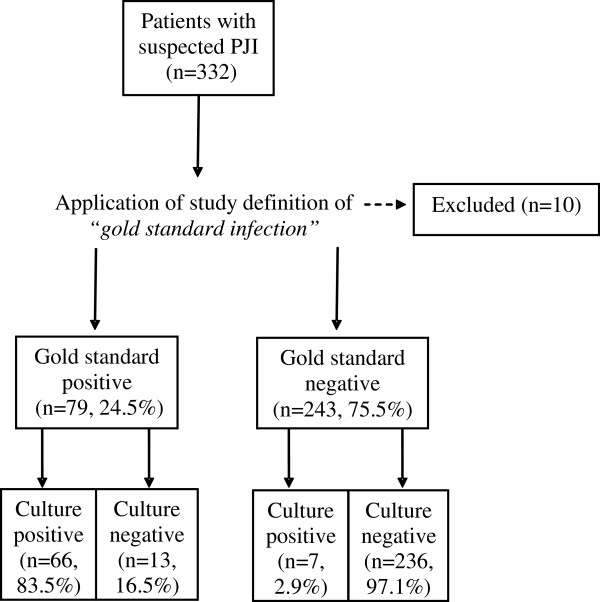
**Flowchart: route to determination of infection (n = no. of patients).** “Culture-positive” refers to patients with indistinguishable isolates from 2 or more samples. *“Gold standard infection”* is defined as one or more histological AND/OR clinical criteria.

Figure 1 shows the numbers of patients involved at initial sampling and subsequent stages through the analytical route to determination of infection. A total of 332 patients with suspected PJI were identified. In 10 cases (all culture negative) there were insufficient data on histology or clinical criteria to ascertain presence or absence of *“gold standard infection”*. These were excluded, leaving 322 patients. 79 (24.5%) of these patients had *“gold standard infection”*, the remaining 243 were classified as being *“gold standard negative”* (Figure 1). Of the 66 culture-positive patients with *“gold standard infection”*, 45 were due to a single organism and 21 were mixed. Of those with mixed cultures, 4/21 (19.0%) had a sinus, compared to 14/45 (31.1%) of those with single cultures (*P* = 0.383, Fisher’s exact 2-sided test).

### Time to positivity (TTP) by species and culture atmosphere

**Figure 2:**
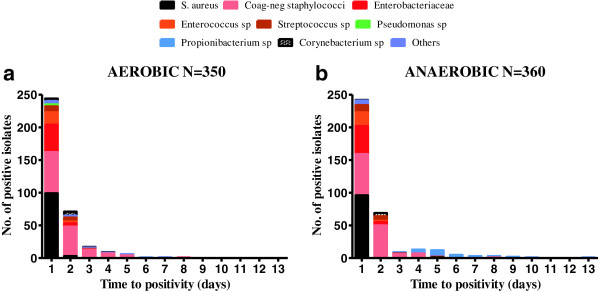
**Time to positivity (TTP) (days) by organism: data for aerobic (a) and anaerobic (b) positive cultures shown separately, for ALL isolates grown from all 1328 periprosthetic samples.** Data include no determination of whether or not *“gold standard infection*”*.* N = 360 means 360 of the 455 isolates flagged in the aerobic bottle; N = 350 means 350 of the 455 isolates flagged in the anaerobic bottle.

The time to positivity of isolates cultured from patients with suspected PJI over the 7-month study period is shown in Figure 2 (and Additional file 1: Table S1). These are categorised into broad groups of species. The commonest organisms were coagulase-negative staphylococci (CNS, 37%), *Staphylococcus aureus* (28%) and *Enterobacteriaceae* (13%). For aerobic cultures, 95% of organisms were detected within 3 days, 100% within 8 days. For anaerobic cultures, 96% were detected within 5 days, and 99% within 10 days.

### Both aerobic and anaerobic culture conditions are crucial

**Figure 3:**
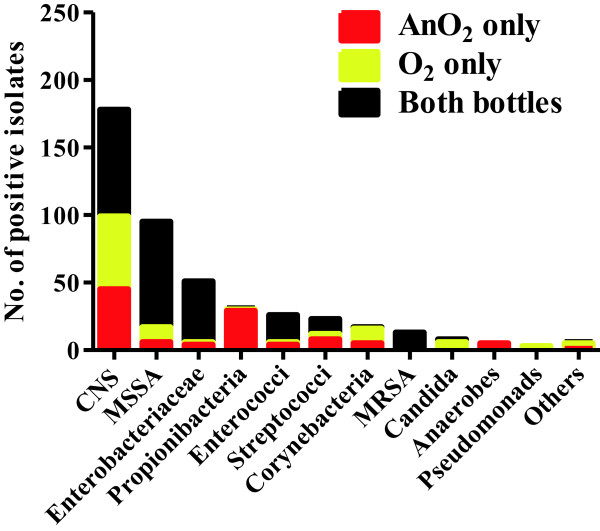
**Number of isolates flagging in one or both BACTEC™ bottles (aerobic, O**_**2, **_**and anaerobic, AnO**_**2**_**), by organism type.** Graph shows that a significant proportion of isolates would remain undetected without dual culture conditions in parallel.

14% of organisms were identified from the aerobic bottle only and 27% from the anaerobic bottle only. Figure 3 shows the number of isolates flagging in one or both BACTEC™ bottles (aerobic, O_2,_ and anaerobic, AnO_2_), by organism type. It demonstrates that restricting the conditions to aerobic alone would result in failure to identify the majority of *Propionibacteria spp.*, anaerobes and many *Streptococcus spp.*, whereas anaerobic-only conditions would result in an apparent absence of *Pseudomonas spp*, *Corynebacteria spp.* and *Candida spp.*

### Sensitivity & Specificity by culture incubation period

**Figure 4:**
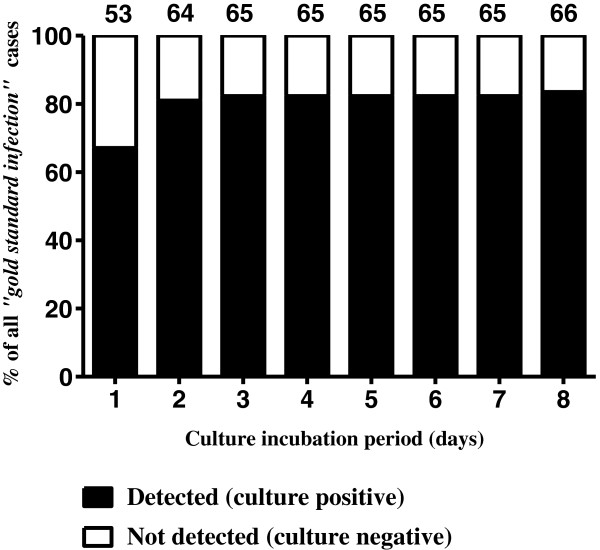
**Sensitivity by culture incubation period, all *“gold standard infection” *cases (N = 79): graph shows the proportion culture positive (solid black bars) among *“gold standard infection” *cases, by incubation period.** Numbers above the bars relate to culture positive cases.

Among the 79 *“gold standard infection”* cases, 66 were culture-positive (i.e. 83.5%) within 8 days of culture. Of the remaining 13 culture-negative cases, six patients were either on antibiotics at the time of sampling or had been on antibiotics within the 10 days prior to sampling. The sensitivity of BACTEC™ for diagnosis of PJI increased from 67.1% at day 1, to 82.3% at 3 days of incubation. Thereafter further incubation of samples failed to increase the sensitivity until a minor increment at 8 days (when sensitivity reached 83.5%), corresponding to the identification of one significant *Propionibacterium acnes* isolate. Details are shown in Table 1 and Figure 4.

**Table 1:** **Sensitivity and specificity of BACTEC**^**TM **^**using “gold standard” definition for infection**

**Incubation period**	***“Gold standard” *** **infection**	**Cumulative number of patients**			**% correctly identified by culture**	
		**Culture + ve**	**Culture -ve**	**Total**	**Sensitivity (95% CI)**	**Specificity (95% CI)**
1 day	Present	53	26	79	67.1 (55.6-77.3)	
	Absent	1	242	243		99.6 (97.7-100)
2 days	Present	64	15	79	81.0 (70.6-89.0)	
	Absent	2	241	243		99.2 (97.1-99.9)
3 days	Present	65	14	79	82.3 (72.1-90.0)	
	Absent	3	240	243		98.8 (96.4-99.7)
4 days	Present	65	14	79	82.3 (72.1-90.0)	
	Absent	4	239	243		98.4 (95.8-99.5)
5 days	Present	65	14	79	82.3 (72.1-09.0)	
	Absent	7	236	243		97.1 (94.2-98.8)
6 days	Present	65	14	79	82.3 (72.1-90.0)	
	Absent	7	236	243		97.1 (94.2-98.8)
7 days	Present	65	14	79	82.3 (72.1-90.0)	
	Absent	7	236	243		97.1 (94.2-98.8)
8 days	Present	66	13	79	83.5 (73.5-90.9)	
	Absent	7	236	243		97.1 (94.2-98.8)

Sensitivity and specificity for each time-point (incubation period) are shown in percentages with 95% confidence intervals. “Culture + ve” refers to patients with 2 or more samples positive with the same organism. “Culture –ve” refers to the rest.

Of the 243 *“gold standard negative”* cases, 236 remained culture-negative throughout (i.e. 97.1% correctly identified by BACTEC™). The specificity at day 1 was 99.6%, remaining high at 98.8% and 97.1% at 3 and 5 days of incubation respectively, with no further deterioration thereafter (Table 1). 189 of the 236 (80.1%) culture-negative cases were also negative for histological criteria, 10 (4.2%) were equivocal and 37 (15.7%) had no samples sent for histological analysis. The remaining 7 *“gold standard negative”* cases were culture-positive but all of these were negative by histological and clinical criteria. 4 of these 7 grew a *Propionibacterium*, 4 a CNS, and 1 a *Bacillus spp.*

### Optimal combination of sensitivity (Sn) and specificity (Sp)

Receiver Operator Characteristics analysis was performed for incubation periods of 1 to 8 days, giving equal weights (importance) to Sn and Sp. The combination of Sn and Sp values maximized at day 3 (i.e., optimal cut-off), with a sensitivity of 82.3% (CI: 72.1-90.0) and specificity of 98.8% (CI: 96.4-99.7). (Additional file 2: Figure S1).

### Using the less stringent criteria for culture positivity

We identified 11 cases where only a single sample was positive for a virulent organism (e.g., *Staphylococcus aureus, E coli, Candida spp).* These cases did not meet our definition for microbiological positivity, but may be judged important in some circumstances. When these cases were also counted as culture-positive, the optimal incubation period was 4 days, with a corresponding sensitivity of 84.3% (CI: 75.0 - 91.1) and specificity of 97.9% (CI: 95.3 - 99.3). The findings for incubation period of 3 days were: Sn 83.15% (CI: 73.7 - 90.3), Sp 98.36% (CI: 95.9 - 99.6).

### Detection of propionibacteria spp

Isolates of *Propionibacteria spp.* were detected at a median of 5 days of culture incubation, compared with a median of 1 day of culture for other bacterial species. 30 isolates of *Propionibacteria spp.* were grown from 67 samples taken from 16 patients over the study time-frame. Only 2 of these 16 patients had *“gold standard infection”*; both fulfilled clinical and histological criteria and were detected by BACTEC™ at 3 and 8 days. 12 patients did not fulfil the criteria for *“gold standard infection”* and for the remaining 2 there was insufficient clinical information.

### Sub-culturing of 14-day negative samples

Of the 1000 non-flagging BACTEC™ bottle cultures selected for terminal subculture at day 14, 987 samples remained culture-negative after a further 5 days’ incubation on FAA and chocolate agar. 12 anaerobic bottles (from 3 patients) were positive for *Pseudomonas aeruginosa*, but this had no clinical impact since the corresponding aerobic bottles had grown the same organism while on the BACTEC™ machine at an earlier time-point. Only one patient’s sample was positive for *Propionibacterium acnes* on terminal subculture. This was a patient who fulfilled our *“gold standard”* definition of PJI. The specimen was 1 of 5 specimens from the same patient, where 3 of the remaining 4 specimens had flagged with a *Propionibacterium* on the BACTEC™ machine at earlier time-points (8, 9 & 10 days’ incubation).

## Discussion

Cultures from deep intraoperative specimens are used to diagnose PJI and to guide antibiotic treatment. Applying a composite definition for PJI (comprising histological and clinical criteria to fulfil a *“gold standard”*), and defining positive microbiology as 2 or more similar isolates from multiple specimens, we have shown that the majority of clinically significant organisms are detected by BACTEC™ within 3 days of incubation. In line with recent recommendations [10], we conducted secondary analyses to include the isolation of 1 virulent organism from a single sample in the definition of a positive microbiological result. Using this more sensitive, but perhaps, less specific definition, an incubation period of 4 days gave an optimal combined sensitivity and specificity.

While we recognize that our *“gold standard”* definition of PJI is imperfect, especially in that it may lack sensitivity, the specificity of the definition is high, and we chose a *“gold standard”* that did not include any bacteriological component in order to avoid circularity in the analysis. Our definition of PJI is also in line with recent guidelines from the Infectious Diseases Society of America [6].

BACTEC™ successfully identified 30 isolates of *Propionibacteria spp.* from 67 samples over the study time-frame. Sub-culturing BACTEC™ bottles which were negative after 14-days’ incubation detected only a single additional *Propionibacterium* isolate from 1000 bottles. Other bottles containing culture material from the same patient had already been detected as positive by BACTEC™, so this additional information had no clinical consequences. Interestingly, Butler-Wu *et al.,* found that only 40% of significant cases of PJI secondary to *Propionibacteria spp.* fulfilled histological criteria for infection (≥5 neutrophils/high-power field). While 4 of our 7 culture-positive cases that were *“gold-standard negative”* for infection (and therefore negative by histological criteria) grew a *Propionibacterium*, both of our “*gold standard positive*” cases of *Propionibacterium*, were in fact positive for histological as well as clinical features of infection. However, these small numbers limit further interpretation.

It is possible that the excellent sensitivity of BACTEC™ and rapid growth observed over the study period were in part due to vortexing with sterile beads, rather than to the BD BACTEC™ incubation *per say.* The use of a beadmill has previously been shown to be highly effective in the microbiological identification of PJI, using multiple peri-prosthetic samples, with inoculation onto solid and liquid media. Prolonging culture incubation from 7 to 14 days also failed to increase the sensitivity of this method [11]. No studies have yet compared the efficacy of BACTEC™ with and without prior beadmill processing.

One unknown capacity of our methodology surrounds the detection of small colony variants (SCVs). SVCs have auxotrophisms for hemin, menadione and thymidine [12], the first two of which are present in the BACTEC™ bottle medium. We are currently doing experiments to address this further, however, this study was not designed to evaluate the role of SVCs in this setting. It is also possible that the sensitivity of instrumented blood culture systems could be improved by sonication of implants prior to culture, although this is not always available in routine laboratories, and may reduce specificity [13].

## Conclusion

In summary, we recommend using an instrumented blood culture system such as BD BACTEC™ as a convenient, simple to use and rapid method for diagnosis of PJI. We have shown that this reliably detects slow-growing organisms such as *Propionibacteria spp.* and that prolonged culture periods are not necessary. These findings should facilitate earlier decisions on final antimicrobial prescribing.

## Competing interests

The authors declare that they have no competing interests.

## Authors’ contributions

AMM participated in the design and coordination of the study, performed the statistical analysis and interpretation of data and wrote the manuscript. RN coordinated the laboratory work and contributed to the writing of the manuscript. EK performed the laboratory work. BLA and ICWB conceived of the study, participated in the design and coordination of the study and contributed to the writing/editing of the manuscript. PB advised on the design of the study and statistical analysis and contributed to the writing/editing of the manuscript. All authors read and approved the final manuscript.

## Pre-publication history

The pre-publication history for this paper can be accessed here:

http://www.biomedcentral.com/1471-2334/14/233/prepub

## Supplementary Material

Additional file 1: Table S1 Time to positivity (TTP) (days) by organism. Click here for file

Additional file 2: Figure S1 Receiver Operator Curve Analysis Plot: optimal combination of sensitivity (Sn) and specificity (Sp) occurs when the difference between Sn and (1-Sp) is maximized, which is represented by the longest perpendicular line from the diagonal line of equality to the curve, cutting the curve at incubation period of 3 days. Click here for file
