# Supplementary material for: Use of an automated blood culture system (BD BACTEC™) for diagnosis of prosthetic joint infections: easy and fast
Source: BMC Infect Dis. 2014 May 4;14:233. doi: 10.1186/1471-2334-14-233 (PMC4101863; doi:10.1186/1471-2334-14-233)
Supplement: Additional file 1: Table S1 — Time to positivity (TTP) (days) by organism. [file 1471-2334-14-233-S1.docx]

**Supplementary Table 1: Time to positivity (TTP) (days) by organism**

| **TTP** | **S. aureus** | **CNS** | **Enterobact** | **Enterococ** | **Strep** | **Pseudo** | **Propi** | **Coryne** | **Others** |
| --- | --- | --- | --- | --- | --- | --- | --- | --- | --- |
| **1** | 99, 96 | 63, 63 | 42, 43 | 19, 21 | 9, 11 | 3, 0 | 0, 0 | 4, 1 | 5, 7 |
| **2** | 3, 0 | 45, 50 | 5, 5 | 3, 2 | 6, 7 | 0, 0 | 0, 0 | 6, 5 | 3, 0 |
| **3** | 0, 0 | 13, 6 | 0, 0 | 0, 0 | 1, 1 | 0, 0 | 0, 1 | 1, 0 | 2, 1 |
| **4** | 0, 0 | 7, 7 | 0, 0 | 0, 0 | 0, 0 | 0, 0 | 0, 6 | 1, 0 | 1, 0 |
| **5** | 0, 1 | 4, 0 | 0, 0 | 0, 1 | 0, 0 | 0, 0 | 1, 9 | 0, 0 | 1, 1 |
| **6** | 0. 0 | 0, 0 | 0, 0 | 0, 0 | 0, 0 | 0, 0 | 1, 5 | 0, 0 | 0, 0 |
| **7** | 0, 0 | 0, 0 | 0, 0 | 0, 0 | 0, 0 | 0, 0 | 0, 3 | 0, 0 | 1, 0 |
| **8** | 0, 0 | 1, 1 | 0, 0 | 0, 0 | 0, 0 | 0, 0 | 0, 2 | 0, 0 | 0, 0 |
| **9** | 0, 0 | 0, 0 | 0, 0 | 0, 0 | 0, 0 | 0, 0 | 0, 2 | 0, 0 | 0, 0 |
| **10** | 0, 0 | 0, 0 | 0, 0 | 0, 0 | 0, 0 | 0, 0 | 0, 1 | 0, 0 | 0, 0 |
| **11** | 0, 0 | 0, 0 | 0, 0 | 0, 0 | 0, 0 | 0, 0 | 0, 0 | 0, 0 | 0, 0 |
| **12** | 0, 0 | 0, 0 | 0, 0 | 0, 0 | 0, 0 | 0, 0 | 0, 0 | 0, 0 | 0, 0 |
| **13** | 0, 0 | 0, 0 | 0, 0 | 0, 0 | 0, 0 | 0, 0 | 0, 1 | 0, 0 | 0, 0 |

Numerical data shown for aerobic isolates (left of column) and anaerobic isolates (right of column) grown from all 1328 periprosthetic samples.

Data include no determination of whether *“gold standard”* definition of PJI met.
