# Supplementary material for: Use of an automated blood culture system (BD BACTEC™) for diagnosis of prosthetic joint infections: easy and fast
Source: BMC Infect Dis. 2014 May 4;14:233. doi: 10.1186/1471-2334-14-233 (PMC4101863; doi:10.1186/1471-2334-14-233)
Supplement: Additional file 2: Figure S1 — Receiver Operator Curve Analysis Plot: optimal combination of sensitivity (Sn) and specificity (Sp) occurs when the difference between Sn and (1-Sp) is maximized, which is represented by the longest perpendicular line from the diagonal line of equality to the curve, cutting the curve at incubation period of 3 days. [file 1471-2334-14-233-S2.docx]

**Supplementary Figure 1**

**Receiver Operator Curve Analysis Plot:** optimal combination of sensitivity (Sn) and specificity (Sp) occurs when the difference between Sn and (1-Sp) is maximized, which is represented by the longest perpendicular line from the diagonal line of equality to the curve, cutting the curve at incubation period of 3 days.
